# Defining and distinguishing infant behavioral states using acoustic cry analysis: is colic painful?

**DOI:** 10.1038/s41390-019-0592-4

**Published:** 2019-10-04

**Authors:** Joanna J. Parga, Sharon Lewin, Juanita Lewis, Diana Montoya-Williams, Abeer Alwan, Brianna Shaul, Carol Han, Susan Y. Bookheimer, Sherry Eyer, Mirella Dapretto, Lonnie Zeltzer, Lauren Dunlap, Usha Nookala, Daniel Sun, Bianca H. Dang, Ariana E. Anderson

**Affiliations:** 1 Children’s Hospital of Pennsylvania, Philadelphia, PA USA; 2 David Geffen School of Medicine, University of California, Los Angeles, CA USA; 3 University of California, Los Angeles, CA USA; 4 Private Practice, Los Angeles, CA USA; 5 Gallaudet University, Washington, DC USA

## Abstract

**Background:**

To characterize acoustic features of an infant’s cry and use machine learning to provide an objective measurement of behavioral state in a cry-translator. To apply the cry-translation algorithm to colic hypothesizing that these cries sound painful.

**Methods:**

Assessment of 1000 cries in a mobile app (ChatterBaby^TM^). Training a cry-translation algorithm by evaluating >6000 acoustic features to predict whether infant cry was due to a pain (vaccinations, ear-piercings), fussy, or hunger states. Using the algorithm to predict the behavioral state of infants with reported colic.

**Results:**

The cry-translation algorithm was 90.7% accurate for identifying pain cries, and achieved 71.5% accuracy in discriminating cries from fussiness, hunger, or pain. The ChatterBaby cry-translation algorithm overwhelmingly predicted that colic cries were most likely from pain, compared to fussy and hungry states. Colic cries had average pain ratings of 73%, significantly greater than the pain measurements found in fussiness and hunger (*p* < 0.001, 2-sample *t* test). Colic cries outranked pain cries by measures of acoustic intensity, including energy, length of voiced periods, and fundamental frequency/pitch, while fussy and hungry cries showed reduced intensity measures compared to pain and colic.

**Conclusions:**

Acoustic features of cries are consistent across a diverse infant population and can be utilized as objective markers of pain, hunger, and fussiness. The ChatterBaby algorithm detected significant acoustic similarities between colic and painful cries, suggesting that they may share a neuronal pathway.

## Introduction

All infants cry to motivate their caregivers to respond to their needs.^1^ As a result, caregivers tend to interpret a baby crying as a signal of distress or need. Infants follow a predictable cry curve with a peak in intensity at around 6–8 weeks, and persistence after 3 months may be considered pathologic.^2^ The ability to distinguish pathological cries in infants using acoustic feature extraction and classification algorithms is validated in the literature; 27 prior studies were able to discriminate pathological infant cries (Down’s syndrome, brain damage, Cri du Chat) with an average accuracy rate of 96.9%.^3^

Acoustic analyses of an infant’s cry could be instrumental in the home setting. Despite caregivers’ best intentions, interpretation of infant cries can be difficult. The perceptions of the listener can be influenced by their sleep habits, mental state, their own physiologic response to the cry, and other sociodemographic factors.^4,5^ Machine learning could offer an objective assessment of the acoustic features of infant cries to translate their behavioral states.^6^ This would contribute significantly to infant care by distinguishing if an infant was experiencing pain or if they were responding to another behavioral state (i.e., hunger or being fussy).

It is not only in the home environment that machine learning could aid in infant care. Clinical care and especially hospital settings focus on mitigation of infant pain. Historically, it was believed that infants were incapable of feeling pain.^7^ However, recent research into the developmental physiology of nociception indicates that the opposite is true. Untreated pain in neonates can leave a lasting neurophysiological footprint associated with decreased brain^8,9^ and body growth,^10^ altered neural connections and organization,^11,12^ poorer cognitive and motor function,^13^ impaired visual–motor integration, and poorer executive functioning skills.^14,15^ To assess pain, providers rely upon rating scales such as the Neonatal Infant Pain Scale,^16^ premature infant pain profile,^17^ Face, Legs, Activity, Cry, and Consolability scale,^18^ and Crying, Oxygenation, vital signs, facial Expression, and Sleeplessness scale,^19^ among others. Most estimates of inter-rater reliability of infant scales are high^16,20,21^ with some studies showing poor agreement across these scales in measurements,^22,23^ suggesting that both clinical factors and the choice of scale may strongly influence the magnitude and the reliability of these pain measurements. In addition to measurement of pain using subjective infant pain scales, smaller-sample studies have found that infants in pain cry differently from infants who are not experiencing pain—with algorithms showing between 74% and 90% accuracy, as discussed further in the Supplementary Material.

These small-sample algorithms were not portable by nature; this leaves room for a universally applicable machine learning program to help home caregivers and medical providers accurately assess infant cry and determine when the infant is experiencing pain vs. another behavioral state. On the basis of finding a quantitative measure of infant cries, we created a free phone app, ChatterBaby^TM^, as an accessible and portable algorithm deployment to predict whether a baby’s cry was due to one of the three behavioral states: pain, hunger, or fussiness. The algorithms were then applied to infant cries where parents reported their infants as having colic. This process simulates an initial clinical visit where the parent has complaints of colic and a workup for conditions like reflux esophagitis or infantile migraine may be initiated and diagnosed. We hypothesized that colic cries would be acoustically similar to pain cries, a finding that would explain and validate caregiver distress regarding caring for an infant with colic.

## Methods

This ChatterBaby study was conducted according to and approved by the UCLA Institutional Review Board (IRB#15-000931). Painful stimuli were defined by needles: routine vaccinations (without analgesia) and elective ear-piercings. Because audio was recorded in the natural environment, infants were in a variety of settings while being recorded, with ambient occurring background noise (adult voices, etc.) using different recording devices (e.g., cell-phones). Full details on data acquisition and statistical methodology are provided in Supplementary Material. In Supplementary Material, we also present a secondary cry detection algorithm that screens out cries from baby neutral/baby laughing/nuisance sounds.

### Data

After quality control, the study population for the primary cry states (Fussy, Hungry, Pain) included 691 infants (36% female) who were between the ages of 0 and 24 months (average age 3 months) for the primary training dataset of pain/hungry/fussy. Approximately 55% of infants’ ages were missing due to the voluntary submission of this variable. In all, 75% of the infants assessed were <6 months of age. All primary cries were from unique episodes and users. The colic population included 64 infants between the ages of 2 days and 4 months, with a median age of 2 months.

**Fig. 1:**
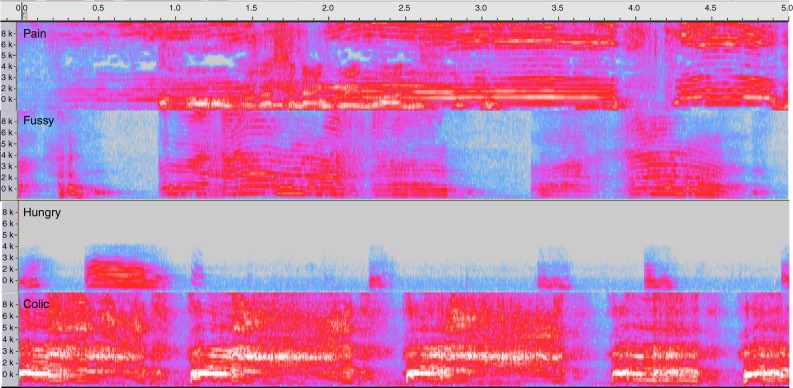
Spectrograms from 5-s audio samples of each cry type showing the distribution of frequencies across time for four different infants. Acoustic features were used to train a machine learning algorithm to predict across three primary cry states: hungry, fussy, pain. This algorithm was tested on infant cries from colic to assess whether acoustic features of pain were present in cries from infants with parental-assessed colic

Pain cries (*n* = 353) were captured during two acutely painful stimuli (vaccinations, ear-piercings). Caretakers characterized other cries as “fussy” and “hungry,” followed by two independent characterization of each cry sound by  two multiparous raters (authors A.E.A. and & B.S.). No cries in the ChatterBaby training database were from any of the authors’ children. Cries without unanimous agreement among the three-member rating panel (11.8%) were excluded from further analyses and were not reclassified. This process resulted in 171 fussy cries, 167 hungry cries, and 353 pain cries in the final training cohort. Colic cries were nominated by the parent/caretaker. Multiple colic cry samples (*n* = 380, 64 babies) were acquired across each cry episode, including ending periods where whimpering/fussing may have been present, to avoid selection bias in sample collection and assessment. This method of data acquisition yielded roughly 30 s (6 samples) of cry time from each child, a process that provided a wider range of time than is typically seen in most studies. Spectrograms for a single cry from each type are presented in Fig. 1.

### Modeling

Infant cries were summarized using the acoustic feature set previously used to identify pathological vocal patterns in neurological disorders such as Parkinson’s Disease^24^ and Amyotrophic Lateral Sclerosis,^25^ extracting >6000 acoustic features from each cry. Supra-segmental (utterance-level) acoustic features were extracted from 5-s cry clips^26–28^ using IS13_ComParE.conf in OpenSmile.^29^

To create the cry translation algorithm, a probabilistic random forests classifier was used to predict the category of a cry (fussy, hungry, pain) given its acoustic features using default parameter settings in R (500 trees, 1/3 of features sampled with replacement as possible predictors to construct individual trees).^30^ The random forests out-of-sample classification accuracy, analogous to the cross-validation error, was computed to estimate the testing accuracy of the algorithm on new data (Table 1). Further technical details are presented in Supplementary Material.

**Table 1:** Predictive accuracy of the random forests classifier for identifying pain cries vs. hungry vs. fussy cries, assessed using the out-of-sample accuracy

Calculated diagnostic accuracy parameters	
Sample size	691
Prevalence	0.51
Sensitivity	0.91
Specificity	0.68
PPV	0.75
NPV	0.87
LR + result	2.81
LR − result	0.14

The primary algorithm was trained on these three cry states that were not developmentally dependent, to assess whether pain ratings differed in babies with colic and without colic. Roughly 51% of cries were painful, but the ChatterBaby algorithm performed significantly above chance and correctly flagged 91% of pain cries

Using only the 200 most predictive features, the algorithm was retrained on the primary cries and tested on the colic cries, with roughly 6 colic cries obtained from the same cry episode per child (~30 s). Testing longer cry segments from the colic infants reduces the probability of selection bias; acoustic sample included segments of milder fussiness and whimpering following extreme bouts of crying, when available. The average pain probability from colic cries was compared with the out-of-sample pain-level predictions from the primary cries (fussy, hungry, pain) to test the hypothesis that colic cries were more closely associated with pain than the hungry or fussy states. We additionally assessed for longitudinal/age effects by testing for temporal drift within a single child who was not used for algorithm training, using cry recordings collected six separate times during routine vaccinations between 87 and 618 days of age, without the usage of analgesic.

## Results

The primary cry algorithm achieved overall accuracy in classifying among the three states as 71.5%, with the confusion matrix shown in Supplementary Material. The primary cry algorithm, trained as a multivariate classifier, was then treated as a binary classifier for obtaining Pain accuracy rates by pooling the Fussy and Hungry predictions as a “No Pain” category. The predictive accuracies for painful cries are shown in Table 1: sensitivity/recall of 0.91 (95% confidence interval (CI) = 0.876, 0.937), specificity = 0.68 (95% CI = 0.628, 0.727), positive predictive value = 0.75, negative predictive value = 0.87. The prevalence of Pain was 0.51, with the algorithm performing significantly above chance (*p* < 0.001). The area under the curve (AUC) = 0.88 as shown in the receiver operating characteristic curve in Supplementary Material. The AUC measures how effective the algorithm is at separating true positives and false positives over a range of decision thresholds.

**Fig. 2:**
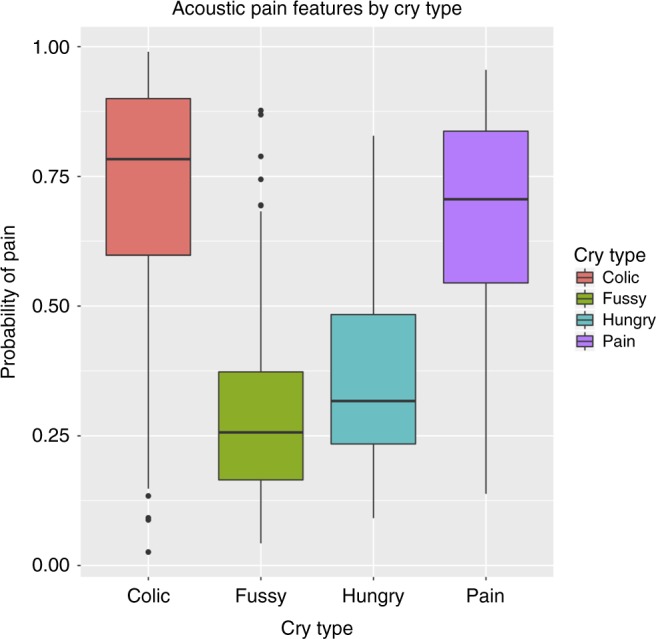
The ChatterBaby algorithms were trained initially using three cry states: Fussy, Hungry, and Pain. The algorithms were validated both internally using the out-of-bag testing accuracy as well as externally; the algorithms were tested on a separate subset of baby cries from Colic (as defined by the parent). Colic cries had significantly higher acoustic measures of Acute Pain compared to Fussy and Hungry (*p* < 0.001)

Although the random forests algorithm had access to >6000 features, many of these features had low importance values and were not useful to discriminate among the different cry states, as shown in Supplementary Material. When testing the algorithm on colic cries, the probability of pain was significantly different across the three predictive states (*p* < 0.0001, analysis of variance), with the typical colic cry being predicted as 73% chance of painful. When comparing colic cries to fussy/hungry cries, the pain levels in colic were significantly greater than the pain levels seen in fussy and hungry cries (*p* < 0.001, 2-sample *t* test, Bonferroni corrected). As shown in Fig. 2, the average pain rating in colic was 0.73 (sd = 0.21), while the average out-of-sample predicted pain rating for fussy was 0.30 (sd = 0.18), hungry = 0.38 (sd = 0.19), and pain = 0.67 (sd = 0.20).

Previous literature demonstrated an increased pitch (fundamental frequency) in both pain and colic^31^ cries compared to fussy and hungry states, which we confirmed here (*p* < 0.05; 2-sample *t* test with Bonferroni correction, see Supplementary Material). The pitch did not significantly differ between colic and pain (*p* > 0.05, 2-sample *t* test with Bonferroni correction; see Supplementary Material). For many acoustic features such as loudness, energy, and pitch, the ordinal values fell in a spectrum ranging from fussy, hungry, pain, to colic. This spectrum suggests that colic cries are more intense acoustically than vaccination cries, although the clinical interpretation of this acoustic relationship is unknown. Fussy cries were the mildest acoustically across many acoustic metrics.

## Discussion

It is possible to use mobile recording methods to provide accurate and usable clinical information on an infant’s cry and behavioral state. With 70–90% accuracy, an easily accessible mobile app was built off of prior knowledge of the acoustical features of pathological cries in infancy. It was used to further explore a common diagnosis of infancy affecting one in five neonates and defined entirely by excessive crying: colic.

The acoustic markers of pain were multiple and complex, extending far beyond changes in pitch as was reported previously in the literature.^31^ The colic cries were not different from pain cries in their fundamental frequency (2-sample *t* test, *p* > 0.05), but the colic fundamental frequency was significantly elevated compared to hungry and fussy vocalizations (*p* < 0.05, Bonferroni corrected). This confirms the earlier findings of Lester et al. and St. James-Robert,^32,33^ which relied on significantly smaller-sample sizes than those assessed here.

Our work demonstrated that colic cries are more similar to pain cries than to either fussy or hungry cries, suggesting that colic could be a painful condition for infants or share similar source processes.^34^ Often colic occurs in the evening and clinicians do not observe it and have to rely on caregivers’ reports of the crying. Positive reinforcement and support for caregivers is considered the standard of clinical care in colic^2^ and focuses on helping caregivers through a stressful period. In 95% of cases of colic, a thorough workup for underlying medical disorders fails to uncover a definitive explanation for the infant’s presentation, and these infants will develop normally once they “outgrow” their colic.

Despite treatment through reassurance from providers, infantile colic is associated with increased rates of maternal anxiety and depression.^35–39^ Our results suggest that parents may be distressed by the cries of infants with colic because they may hear acoustic signatures indicative of pain as demonstrated in the algorithm. As such, clinicians might consider pain control (i.e., appropriate Tylenol dosing or behavioral pain control methods) as part of the management of colic.

There are several limitations to this study. Not all infants may respond to pain with a cry, thus a subset of infants experiencing pain may not have been reviewed. Our pain cries were in response to acutely painful stimuli; chronic pain may not show the same acoustic features. This could be elucidated with more pain samples from infants experiencing chronic pain (i.e., hospitalization with need for multiple procedures, such as intravenous access and lumbar punctures). Pain is also a subjective feeling, and degree of pain experienced by infants in the study could not be assessed. In addition, colic cries were labeled using parental assessment. It is not known whether these infants carried a clinical diagnosis of colic or whether they ever underwent any treatment for underlying medical conditions. Future studies will focus on clinically determined colic, rather than relying solely upon parental assessment. Of note, the diagnosis of colic is often based on history; so despite this being a limitation, it is likely a technique used diagnostically in the pediatrician’s office. The environment of the data collection was varied because it was performed by the caretakers: infants were in a variety of positions while vocalizing with naturalistic background noises present including adult voices and small children and were collected using a variety of recording devices such as cell-phones. However, the absence of a controlled environment simulates the variability of the testing environment in which these algorithms ultimately will be used, providing a more realistic estimate than previously published work on how these algorithms will fare when applied to new infants in new environments. Finally, we did not optimize the machine learning parameters within this algorithm intentionally, in order to avoid biasing the testing accuracy estimate. Our results are likely a lower bound for predictive accuracy, which we will refine with new data using deep learning algorithms.

**Fig. 3:**
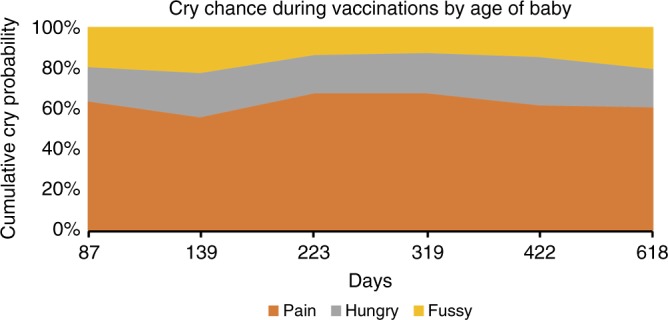
For a single infant, we compared cry recordings during vaccinations for six different events ranging between 87 and 618 days of age. Each vaccination cry was analyzed using the ChatterBaby cry-translation algorithm. For all events, pain had the largest predicted probability and varied little across time in probability. This may suggest that, within a single infant, the vocal features of pain may be highly consistent, which enables parents to learn their child’s cry

Cry profiles may differ by age, which was unlikely to affect our results in secondary testing. Within the longitudinal vaccination recordings from a single child who was not used to create the algorithm, the Fussy/Hungry/Pain algorithm predicted similarly and consistently that the baby was experiencing pain for all six trials (average pain probability = 0.63, sd = 0.04). This suggests that the algorithm was not sensitive to aging effects within the age range evaluated (Fig. 3, also see Supplementary Material for Spectrograms of vaccine cry across age). Five-s audio clips from this child’s vaccinations over an 18-month period are available online at https://www.youtube.com/watch?v=eu332YZFTkA. Infant age and demographics were voluntarily provided, resulting in missing data. Because of this, we could not determine whether the predictive accuracy of our algorithm depends upon an infant’s age or whether our algorithm performs differently on preterm infants or those with developmental disorders. However, for a single infant not contained in the algorithm training dataset, six vaccination cry recordings were examined for age-related variation in pain ratings. These cry recordings were taken between 87 days and 618 days. Overall, the cry patterns were consistent across age (Fig. 3), but because this was for a single child, we cannot rule out different growth patterns in other children.

## Conclusion

Although infant pain has both short- and long-term consequences, previously there was no automated quantitative device for pain or behavioral assessment in the home environment where most crying occurs. We developed a solution as a free smartphone app, ChatterBaby, available at https://chatterbaby.org. The measurements derived from the ChatterBaby algorithm may have in-hospital functions as well- a direction for future research. Passive acoustic pain assessment could serve as a complement to infant pain scales or a baseline metric for comparison of existing infant pain scales. With machine learning, we explored the acoustical features of excessive crying or colic. Future work will explore further evidence of whether colic is painful or whether colic merely shares similar neuronal connections as pain sensations. Such distinction would identify whether  pain control merits as a part of colic treatment. The benefits and utility of a cry-translation algorithm have yet to be executed in clinical practice but are promising and wide-reaching, meriting further investigation.

## Supplementary information

Supplementary Appendix
