# Supplementary material for: Defining and distinguishing infant behavioral states using acoustic cry analysis: is colic painful?
Source: Pediatr Res. 2019 Oct 4;87(3):576–80. doi: 10.1038/s41390-019-0592-4 (PMC7033040; doi:10.1038/s41390-019-0592-4)
Supplement: Supplementary file 1 — Supplementary Appendix [file 41390_2019_592_MOESM1_ESM.docx]

**APPENDIX:**

*Background:*

Infant crying activates distinct brain regions in both mothers[1, 2] and fathers[3], showing that parents are wired to be responsive to a baby’s needs. The ChatterBaby project was originally designed as a baby monitor for Deaf parents, using machine learning and signal processing algorithms to identify whether and why a baby is crying. These algorithms are embedded into free smart-phone apps to translate a baby’s cry into three primary states described below, provided for parents and caretakers (ChatterBaby.org, Figure 1). The app also functions as a data collection device, which will map cry features to developmental disabilities such as autism spectrum disorder which affects up to 1 in 25 children who are Deaf. [4]

Once subjects download the app, they consent to be in the IRB-approved research study and are followed for six years. Developmental questionnaires and autism screening instruments are administered by email each year on the child’s birthday, using validated instruments such as the CDC 12-month Milestones [5], the Quantitative Checklist for Autism in Toddlers (Q-CHAT) [6], and the Childhood Asperger Syndrome Test or the Social and Communication Development Questionnaire (CAST) [7]. The CAST is a screening questionnaire for autism spectrum conditions in children ages 4-11 [7]. RedCap is used for administering all screening instruments, which permits automation of all processes. Beginning at age 2, risk scores for ASD are calculated using the Q-CHAT and CAST scoring systems, and these scores are provided to the participant.

To collect infant cry data for later research, ChatterBaby performs a free service to parents by predicting the reason of a baby’s cry, using data previously labeled by a “mom-panel” or obtained during painful procedures (vaccinations, ear-piercings). Most estimates of inter-rater reliability of infant scales are high [8-10] with some divergence [11]. While some studies showed agreement[12], other studies showed poor agreement across these scales in measurements [13, 14], suggesting that both clinical factors and the choice of scale may strongly influence the magnitude and the reliability of these pain measurements. An objective clinical marker may provide a benchmark for comparing the results of these instruments, with acoustic analyses providing small-sample studies demonstrating feasibility of such a vocal pain metric.

*Supplemental Methods:*

This study was conducted according to and approved by the UCLA Institutional Review Board. Because audio was recorded in the home environment, infants were in a variety of settings while being recorded, with naturally occurring background noise (adult voices, etc.) using different recording devices (e.g., cell-phones). All recordings were converted into 5 second raw pulse-code modulation (PCM), sampling rate 16000 Hz, quantization of 16 bits, and were part of a larger infant vocalization database discussed further in the Appendix. All cry audio recordings were 5 seconds long with the exception of 21 separation anxiety cries which were <3 seconds in length collected by the Oxford Vocal (OxVoc) Sounds database[15].

*Modeling:*

Supra-segmental (utterance-level) acoustic features were extracted from 5 second cry clips [16-18] using IS13_ComParE.conf in OpenSmile [19]. This feature set has been used previously to assess pathological vocal patterns in neurological disorders such as Parkinson’s Disease [20] and Amyotrophic Lateral Sclerosis [21], and contains 6,373 static features computed from various functionals over low-level descriptor (LLD) contours. The pre-defined acoustic features computed various statistics of jitter, shimmer, MFCCs, and spectral features described in more depth in the documentation [19]. A probabilistic random forests classifier was used to predict the category of a cry (fussy, hungry, pain) given its acoustic features using default parameter settings in R (500 trees, 1/3 of features sampled with replacement as possible predictors to construct individual trees). Parameter tuning was avoided to obtain an unbiased estimate of testing accuracy. The random forests model (package randomForest in R) uses resampling of both features and data to create decision tree models, where voting over many decision trees determines an observation’s final class [22]. The random forests out-of-sample classification accuracy, analogous to the cross-validation error, was computed to estimate the testing accuracy of the algorithm on new data. Secondary analyses using parameter tuning showed marginal improvement in the random Forests algorithm, and similar results were realized using other untuned machine learning algorithms (support vector machines) using traditional cross-validation approaches.

*External Validation on Secondary Cries:*

The random forests algorithm provides out-of-sample predictions for observations (out-of-bag estimates), analogous to a cross-validation procedure in other machine learning algorithms. This provides an estimate of how, for example, a new fussy cry performs when using the 3-way classification algorithm. These out-of-sample fussy and hungry states provided control conditions for colic, to assess whether pain levels are increased for infants who are described by caregivers as having colic.

The feature importance values of the algorithm provide a measure of the predictive ability of each feature, and decreased sharply after roughly 100 features (Figure 2). Using only the 200 most predictive features, the algorithm was retrained on the primary cries and tested on the colic cries, with roughly 6 colic cries obtained from the same cry episode per child. The average pain probability from colic cries were compared with the out-of-sample pain predictions from fussy, hungry, and pain to test the hypothesis that colic cries were more closely associated with pain than the hungry or fussy states.

*Analyses of Reduced Feature Sets: the value of extensive feature extraction*

Pain cry acoustics literature has relied heavily on a limited number of acoustic descriptors such as pitch and energy. We assessed the value of the additional acoustic features by extracting a reduced feature set containing 62 acoustic features in the OpenSmile GeMAPSv01a.conf file by Eyben and colleagues[23] . The F0 (pitch) values, extracted with GeMaps in OpenSmile, were tested to assess whether the pitch of colic and pain were similar, and whether the pitch of these states were different from the fussy and hungry states.

*Supplemental Results:*

The primary cry algorithm achieved overall accuracy in classifying among the three states as 71.5%, with the confusion matrix shown in the Appendix, Table 3. The primary cry algorithm, trained as a multivariate classifier, was then treated as a binary classifier for obtaining Pain accuracy rates by pooling the Fussy and Hungry predictions as a “No Pain” category. The predictive accuracies for painful cries are shown in Table 1: sensitivity/recall of .91 (95% CI = .876, .937), specificity = .68 (95% CI = .628, .727), positive predictive value = .75, negative predictive value = .87. The prevalence of Pain was .51, with the algorithm performing significantly above chance (p<0.001). The AUC = .88 is shown in the ROC curve in Figure 3.

Within the longitudinal vaccination recordings from a single child who was not used to create the algorithm, the Fussy/Hungry/Pain algorithm predicted similarly and consistently that the baby was experiencing pain for all six trials (average pain probability = .63, sd =.04). This suggests that the algorithm was not sensitive to aging effects within the age range evaluated (Figure 4, Supplemental Figure 4 for Spectrograms of vaccine cry across age). Five-second audio clips from this child’s vaccinations over an 18 month period are available at <https://www.youtube.com/watch?v=eu332YZFTkA>

Although the random forests algorithm had access to over 6,000 features, many of these features had low importance values and were not useful to discriminate among the different cry states, as shown in Figure 5. The top 10 acoustic features most useful for classification are listed in Table 4 Appendix. The top 200 features were used to train the reduced algorithm for validation on the secondary cry states.

When testing the algorithm on Colic cries, the algorithm demonstrated that the probability of pain was significantly different across the three states (p<0.0001, ANOVA), and the pain levels in colic were significantly greater than the pain levels seen in fussy and hungry states (p<0.001, 2-sample t-test, Bonferroni corrected). As shown in Figure 5, the average pain rating in colic was 0.73 (sd = 0.21), while the average out-of-sample predicted pain rating for fussy was 0.30 (sd = 0.18), hungry = .38 (sd = 0.19), and pain = 0.67 (sd = 0.20).

Our comparative analyses of the OpenSmile GeMaps feature set found a roughly 1% increase in classification accuracy using the full IS13_ComParE.conf features compared to the reduced feature set, with features unique to the IS13_ComParE feature set outranking those in the restricted feature set. This suggests that the higher-order features may be aiding in the classification accuracy observed.

Previous literature demonstrated an increased pitch (fundamental frequency) in both pain and colic [24] cries compared to fussy and hungry states, which we confirmed here (p<0.05; 2-sample t-test with Bonferroni correction, Supplemental Table 5). The pitch did not significantly differ between colic and pain (p>0.05, 2-sample t-test with Bonferroni correction). However, elevated pitch was not specific to either pain or colic, and was also seen in baby cries from scared and separation anxiety states when compared to samples from older children. Moreover, pitch was not one of the 200 most important features in the predictive algorithm; the most important predictive feature in the algorithm was a summary statistic of spectral flux, measuring how different one audio frame was from the next, suggesting that the regularity of a cry provided much of the classification power as to the type of cry. This suggests that the acoustic features of pain extend far beyond pitch. Measures of energy and silence did show important variability across cry profiles (Figure 6).

**Cry detection algorithm:**

The Why-Cry algorithm in the ChatterBaby app is embedded within a primary Cry-detection algorithm trained using n=2,346 audio samples, comparing 1,192 Cries to 1,154 distractor states which were recommended by our Deaf focus group. In addition to fussy, hungry, colic, and pain, cry samples attributable to secondary stimuli were also collected taken during a single episode of crying: scared (n=161, 89 babies) and separation anxiety (n=36, 23 babies). Scared, colic, and separation anxiety labels were provided by the parent or primary caretaker; examples of Scared stimuli included “Daddy singing,” “bear,” and “loud noise” as labeled by the participant. A total of new 15 separation anxiety cries were collected and combined with 21 separation anxiety cries provided in Parsons et al. [15], which were acquired when the infants were separated from their primary caregiver (mean age = 6.7 months, *sd* = 0.9 months). The Cry states pooled all six cry conditions described above: Hungry, Fussy, Pain, Separation Anxiety, Scared, Colic.

Our Deaf focus group had reported that conventional cry detectors were triggered by non-cry sounds, so the algorithm trained on Cry vs. Distractor States. The Distractor states included baby voices (80%) as well as nuisance noises which included animal sounds (cats, dogs) [15], adult males and females laughing [15], adult females and males speaking, three year old children speaking, doorbells, phones ringing, and electronic baby toys collected manually as shown in Table 1. Approximately 80% of Distractor states were from infant noises (laughing, neutral). Of the baby laugh/neutral/cry sounds collected, 79.1% of these were from unique speakers.

Similar to before, the random forests algorithm predicted “Cry” vs Distractor States. This algorithm achieved overall 85% accuracy, with AUC = .92 as shown in Figure 2. The sensitivity = .82 (95% CI = .80, .84) with specificity = .88 (95% CI = .86, .90), as shown in Table 2 and Figure 7.

| **Source** | **n** |
| --- | --- |
| 3 year old female speaking | 16 |
| 3 year old male speaking | 7 |
| adult female laughing | 15 |
| adult female speaker | 26 |
| adult male laughing | 15 |
| adult male speaker | 25 |
| Baby neutral (babbling, cooing) | 728 |
| Baby laughing | 209 |
| Baby toys | 72 |
| Cat Meow | 15 |
| Dog Bark | 15 |
| Doorbell | 6 |
| Phone ringing | 5 |

**Table 1:** Distractor states (n=1154) for the Cry Detection algorithm were collected from a variety of household ambient noise sources which were reported by our Deaf focus group to have generated false positives in conventional cry-detection devices. The Distractor states were compared to 1,192 baby cries to create a Cry Detection algorithm.

**Table 2: Accuracy values for Cry Detection algorithm.** This algorithm predicted baby cries from a variety of distractor states: baby laughing/neutral, animal noises (dog, cat), adults speaking and laughing, 3-year-old speech, and household noises (phones, doorbells). 80% of all distractors were from baby sounds, while the remaining 20% were from the noise sources recommended by our Deaf focus group.

**
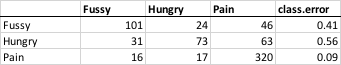
**

**Table 3: Classification accuracy from 3-way classifier.**

**Table 4: Feature Importance of WhyCry algorithm: 10 most predictive acoustic features**

**Table 5:** Acoustic summary statistics by cry type using GeMaps feature set

**
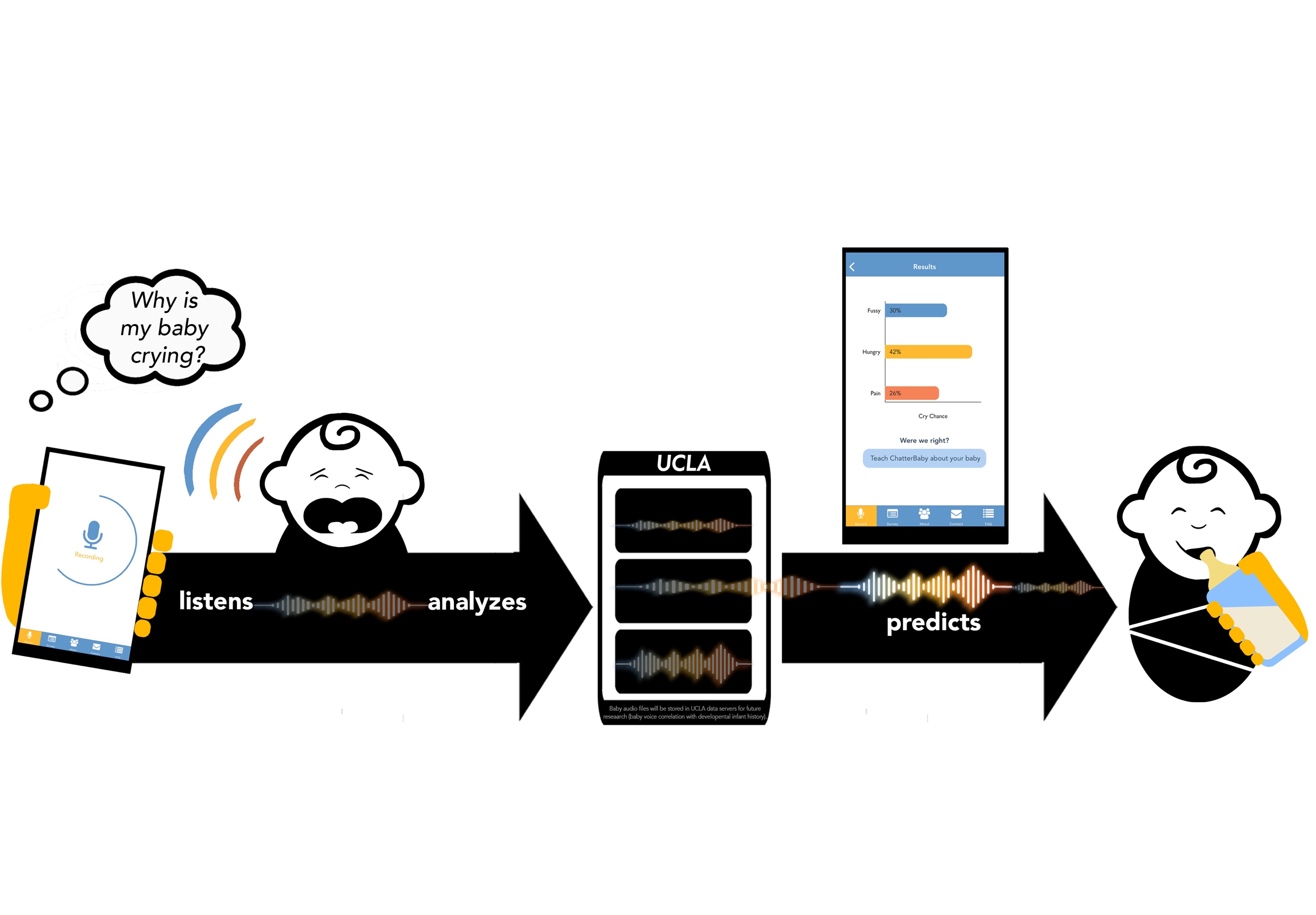
**

**Supplemental Figure S1:** The free ChatterBaby app predicts the most likely reason for a baby’s cry using machine learning. Although originally designed for Deaf parents, this app has shown tremendous international uptake and is used to harvest autism research data from children around the world. Future work on ChatterBaby will assess whether the combination of acoustical biomarkers, phenotypic, and behavioral information may help improve autism screening in children.

**Supplemental Figure S2:** Although over 6000 features were used for classification, many of these had low importance in the random forests machine learning algorithm.

**
Supplemental Figure S3:** Predictive accuracy of algorithm to identify painful infant cries. AUC= .877. Accuracy was defined with respect to the out-of-bag testing accuracy in the random forests algorithm, which is analogous to the cross-validation error. The curve represents the change in the true and false positive rate over a range of probabilistic thresholds used to define a cry as painful.

**Supplemental Figure S4:** As the probability of Pain in a cry increased, the probability of Fussy decreased.

**Supplemental Figure S5:** 11 second Spectrogram of vaccination cries for a single male child aged 87-618 days. Five-second audio clips from this child’s vaccinations over an 18 month period are available at <https://www.youtube.com/watch?v=eu332YZFTkA>

**
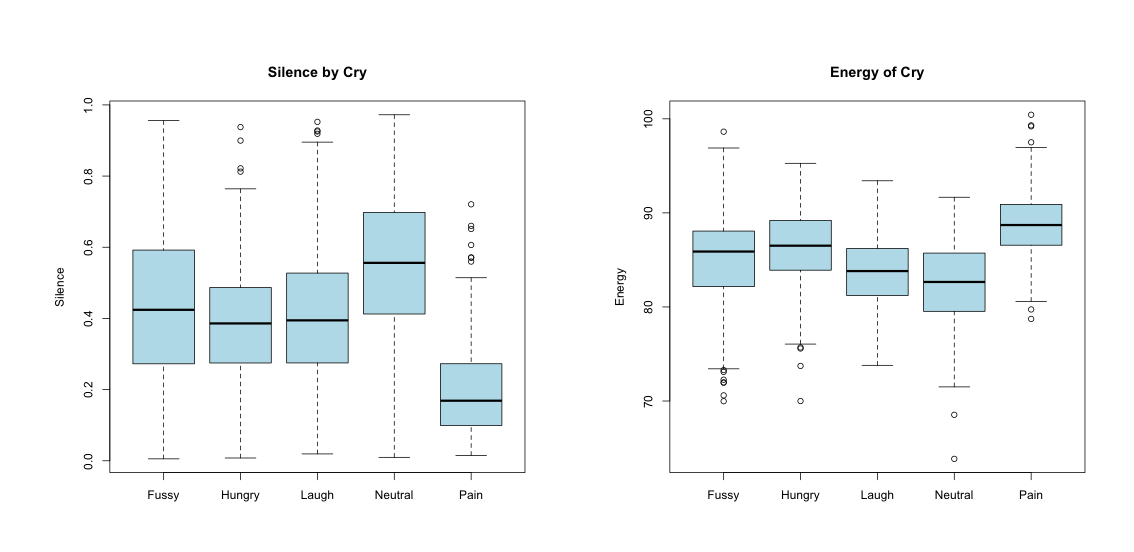
**

**Figure 6:** Cries from babies in pain had more energy and fewer periods of silence.

**Figure 7:** ROC Curve for cry detection. AUC = .92. The 1,152 Distractor states were compared to 1,192 baby cries to create a Cry Detection algorithm.

1. Bornstein, M.H., et al., *Neurobiology of culturally common maternal responses to infant cry.* Proceedings of the National Academy of Sciences, 2017. **114**(45): p. E9465-E9473.

2. Kim, P., et al., *Breastfeeding, brain activation to own infant cry, and maternal sensitivity.* Journal of child psychology and psychiatry, 2011. **52**(8): p. 907-915.

3. Truzzi, A., et al., *Infant communicative signals elicit differential brain dynamics in fathers and non-fathers.* Early Child Development and Care, 2018: p. 1-9.

4. Hitoglou, M., et al., *Childhood autism and auditory system abnormalities.* Pediatric Neurology, 2010. **42**(5): p. 309-314.

5. Control, C.f.D. and Prevention, *Learn the signs. Act early: Developmental milestones*. 2011.

6. Allison, C., et al., *The Q-CHAT (Quantitative CHecklist for Autism in Toddlers): a normally distributed quantitative measure of autistic traits at 18–24 months of age: preliminary report.* Journal of autism and developmental disorders, 2008. **38**(8): p. 1414-1425.

7. Scott, F.J., et al., *The CAST (Childhood Asperger Syndrome Test) Preliminary development of a UK screen for mainstream primary-school-age children.* Autism, 2002. **6**(1): p. 9-31.

8. Jonsdottir, R.B. and G. Kristjansdottir, *The sensitivity of the premature infant pain profile–PIPP to measure pain in hospitalized neonates.* Journal of evaluation in clinical practice, 2005. **11**(6): p. 598-605.

9. Lawrence, J., et al., *The development of a tool to assess neonatal pain.* Neonatal network: NN, 1993. **12**(6): p. 59-66.

10. Taddio, A., et al., *Evaluation of the reliability, validity and practicality of 3 measures of acute pain in infants undergoing immunization injections.* Vaccine, 2011. **29**(7): p. 1390-1394.

11. Bellieni, C.V., et al., *Inter-observer reliability of two pain scales for newborns.* Early human development, 2007. **83**(8): p. 549-552.

12. Ge, X., et al., *Bayesian estimation on diagnostic performance of Face, Legs, Activity, Cry, and Consolability and Neonatal Infant Pain Scale for infant pain assessment in the absence of a gold standard.* Pediatric Anesthesia, 2015. **25**(8): p. 834-839.

13. Ahn, Y., H. Kang, and E. Shin, *Pain assessment using CRIES, FLACC and PIPP in high-risk infants.* Journal of Korean Academy of Nursing, 2005. **35**(7): p. 1401-1409.

14. Suraseranivongse, S., et al., *A comparison of postoperative pain scales in neonates.* BJA: British Journal of Anaesthesia, 2006. **97**(4): p. 540-544.

15. Parsons, C.E., et al., *Introducing the Oxford Vocal (OxVoc) Sounds database: a validated set of non-acted affective sounds from human infants, adults, and domestic animals.* Frontiers in psychology, 2014. **5**: p. 562.

16. Schuller, B., et al., *The INTERSPEECH 2013 computational paralinguistics challenge: social signals, conflict, emotion, autism.* 2013.

17. Schuller, B.W., et al. *The INTERSPEECH 2014 computational paralinguistics challenge: cognitive & physical load*. in *INTERSPEECH*. 2014.

18. Schuller, B., et al. *The INTERSPEECH 2015 Computational Paralinguistics Challenge: Nativeness, Parkinson's & Eating Condition*. in *Sixteenth Annual Conference of the International Speech Communication Association*. 2015.

19. Eyben, F., M. Wöllmer, and B. Schuller. *Opensmile: the munich versatile and fast open-source audio feature extractor*. in *Proceedings of the 18th ACM international conference on Multimedia*. 2010. ACM.

20. Grósz, T., et al. *Assessing the degree of nativeness and Parkinson's condition using Gaussian processes and deep rectifier neural networks*. in *Sixteenth Annual Conference of the International Speech Communication Association*. 2015.

21. Wang, J., et al. *Predicting intelligible speaking rate in individuals with amyotrophic lateral sclerosis from a small number of speech acoustic and articulatory samples*. in *Workshop on Speech and Language Processing for Assistive Technologies*. 2016. NIH Public Access.

22. Breiman, L., *Random forests.* Machine learning, 2001. **45**(1): p. 5-32.

23. Eyben, F., et al., *The Geneva minimalistic acoustic parameter set (GeMAPS) for voice research and affective computing.* IEEE Transactions on Affective Computing, 2016. **7**(2): p. 190-202.

24. Barr, R.G., et al., *Prolonged and unsoothable crying bouts in infants with and without colic.* Journal of Developmental & Behavioral Pediatrics, 2005. **26**(1): p. 14-23.
